# The Functional Mechanism of BP9 in Promoting B Cell Differentiation and Inducing Antigen Presentation

**DOI:** 10.3390/vaccines12060607

**Published:** 2024-06-01

**Authors:** Jianing Hu, Ze Zhang, Jiaxi Cai, Shanshan Hao, Chenfei Li, Xiuli Feng

**Affiliations:** 1 Key Laboratory of Animal Microbiology of China’s Ministry of Agriculture, College of Veterinary Medicine, Nanjing Agricultural University, Nanjing 210095, China; 2021207047@stu.njau.edu.cn (J.H.);; 2 MOE Joint International Research Laboratory of Animal Health and Food Safety, College of Veterinary Medicine, Nanjing Agricultural University, Nanjing 210095, China

**Keywords:** BP9, B cell, high-throughput sequencing, HD11 cell, antigen presentation

## Abstract

The Bursa of Fabricius, an avian unique humoral immune organ, is instrumental to B cell development. Bursal-derived peptide BP9 fosters B-cell development and formation. Yet, the exact mechanism wherein BP9 impacts B cell differentiation and antigenic presentation remains undefined. In this paper, B cell activation and differentiation in the spleen cells from mice immunized with the AIV vaccine and BP9 were detected following flow cytometry (FCM) analysis. Furthermore, the molecular mechanism of BP9 in B cell differentiation in vivo was investigated with RNA sequencing technology. To verify the potential functional mechanism of BP9 in the antigenic presentation process, the transcriptome molecular basis of chicken macrophages stimulated by BP9 was measured via high-throughput sequencing technology. The results proved that when given in experimental dosages, BP9 notably accelerated total B cells, and enhanced B-cell differentiation and plasma cell production. The gene expression profiles of B cells from mice immunized with 0.01 mg/mL BP9 and AIV vaccine disclosed that 0.01 mg/mL BP9 initiated the enrichment of several biological functions and significantly stimulated key B-cell pathways in immunized mice. Crucially, a total of 4093 differentially expressed genes were identified in B cells with BP9 stimulation, including 943 upregulated genes and 3150 downregulated genes. Additionally, BP9 induced various cytokine productions in the chicken macrophage HD11 cells and activated 9 upregulated and 20 downregulated differential miRNAs, which were involved in various signal and biological processes. Furthermore, BP9 stimulated the activation of multiple transcription factors in HD11 cells, which was related to antigen presentation processes. In summary, these results suggested that BP9 might promote B cell differentiation and induce antigen presentation, which might provide the valuable insights into the mechanism of B cell differentiation upon bursal-derived immunomodulating peptide stimulation and provide a solid experimental groundwork for enhancing vaccine-induced immunity.

## 1. Introduction

Given the worldwide threat posed by growing influenza variants, it is imperative to attain comprehensive prevention and control of different influenza virus types [1]. This necessitates urgent strategies, which elicit a robust and enduring immune protective response. The Bursa of Fabricius (BF) serves as a fundamental hub for avian humoral immunity, which plays a critical role in avian B-cell differentiation and maturation [2]. As chicken growth, BF exhibits an ongoing refinement in its tissue structure, characterized by the compact cellular arrangement, the enhanced lymphoid follicle volume, a surge in lymphocyte count, and a steady developmental progression [3]. Nandor et al. highlight the early-phase appearance of the CXCR4 ligand, CXCL12, throughout the BF development process, revealing its pivotal role in B cell development within BF [4]. However, the exact processes guiding the impact of BF on B-cell development and maturation warrant further investigation.

BF is indispensably significant in immunological functions, and a surge in scientific interest focusing on the identification of active molecules and functional mechanisms of BF has thus resulted. One such active component is bursin (Lys-His-Gly-NH2), a BF-derived tripeptide implicated in B-cell development [5]. Bursin as a vaccine adjuvant enhances H9N2 influenza vaccine’s antibody titers while maintaining a balanced Th1 and Th2 immune response and promotes CD4+ and CD8+ T-cell proliferation [6]. Furthermore, BF contains several biologically active substances, which are beneficial for B-cell differentiation. Bursopentin (BP5) induces B cell formation, and promotes B-cell differentiation [7], and attenuates the immune function of dendritic cells [8]. The bursal heptapeptide (BP7) stimulates the strong antibody responses and cell-mediated immune responses and induces 13 signaling pathways and various immune-related functional processes in mouse B cells [9]. Additionally, as an anti-steroidal hormone, the bursal anti-steroidal peptide (BASP) also has the regulatory function on DNA synthesis in bursal lymphocytes and regulating cell differentiation and proliferation [10,11]. The bursal hexapeptide inhibits tumor cell proliferation via p53 signaling [12], and some novel peptide has been reported to promote an immune response to avian immune system developments [13]. Consequently, these data suggest that a plethora of bioactive peptides within the BF may be capable of enhancing vaccine immune responses and B-cell differentiation.

The bursal active peptide BP9 (Leu-Met-Thr-Phe-Arg-Asn-Glu-Gly-Thr-NH2) exhibits the strong inducing roles in antibody response, B-cell differentiation, and autophagy in immature B cells, which provides the link between humoral immunity and B cell differentiation [14]. Also, BP9 augments amplifies the expression of sIgM in avian immature B cells [15]. However, the functional mechanism of BP9 in B cell activation in vivo and antigen presentation is still unclear.

In this paper, the effect of BP9 on splenic lymphocyte differentiation and the molecular mechanism in B-cell differentiation were scrutinized using RNA sequencing technology. Concurrently, the function and molecular mechanism of BP9 in avian macrophage HD11 cells were explored. Building upon prior research, this paper seeks to clarify the mechanisms through which this innovative bursal peptide intensifies immune protection against H9N2 avian influenza vaccines, with particular attention to humoral immune responses and B-cell function. Crucially, the research grants significant insights into the key mechanisms of the humoral immune system, offering a template for clinical tactics in disease prevention and control.

## 2. Materials and Methods

### 2.1. Peptides, Antigens, and Cells

The bursal peptide BP9 (Leu-Met-Thr-Phe-Arg-Asn-Glu-Gly-Thr-NH2) was synthesized with a purity of over 98% from Nanjing GenScript Biotechnology Co., Ltd. (Nanjing, China). The H9N2 strain (A/chicken/Shandong/lY1/2017) of avian influenza virus was maintained in-house [16]. The virus was incubated in 9-day-old SPF chicken embryos, and the hemagglutination titer of the virus was observed to be 2^9^. HD11 cells (chicken macrophages) were donated by the Veterinary Infectious Disease Laboratory of Yangzhou University (Yangzhou, China). Splenic cells, lymphocytes, and HD11 cells were all cultured using DMEM medium containing 10% fetal bovine serum (FBS, FSP500, EXCEL, Suzhou, China).

### 2.2. Experimental Animals

Six-week-old female ICR mice, purchased from the Yangzhou University’s Animal Center (Yangzhou, China), were reared under specific pathogen-free (SPF) conditions.

### 2.3. Main Reagents

Goat anti-mouse IgG secondary antibodies labeled with HRP were purchased from Hangzhou Multi Sciences Biotechnology Company Limited (Hangzhou, China). Lymphocyte isolation was purchased from Beijing Solarbio Science and Technology Company Limited (Beijing, China). Trizol, RT reverse transcription kit, and TB real-time fluorescent quantification kit were purchased from Beijing Takara Biomedical Technology Company Limited (Beijing, China). Bovine serum albumin (BSA), dimethyl sulfoxide (DMSO), MTT, Design and Synthesis of PCR Primers were purchased from Shanghai Sangon Biotech Company Limited (Shanghai, China).

### 2.4. Main Instruments and Equipment

The HW0301-VBA model CO_2_ incubator was purchased from Shanghai Thermo Fisher Scientific Company Limited (Shanghai, China). The TDZ4-WS model low-speed benchtop centrifuge was purchased from Hunan XiangYi Laboratory Instrument Development Company Limited (Xiangyi, China). The ZDX-35 BI model automatic electric pressure steam autoclave was purchased from Shanghai ShenAn Medical Equipment Factory (Shanghai, China). The C6 flow cytometer was purchased from American BD Biosciences Company Limited (Ann Arbor, MI, USA). The BCM-1000A biological clean bench was purchased from Suzhou Antai Airtech Company Limited (Suzhou, China).

### 2.5. Vaccine Preparation and Animal Immunization Protocol

The AIV vaccine was inactivated with 0.1% formaldehyde solution at 37 °C for 24 h, and it was formulated with ISA206 in a 1:1 volume ratio to prepare the immune antigen. Vaccine efficacy was verified based on criteria including vaccine formulation (oil in water emulsion), stability (absence of precipitates), viscosity, and sterility test results.

Six-week-old female mice were distributed into five groups of ten, which were immunized with different immunization treatments, namely PBS (pH = 7.4), AIV vaccine, AIV vaccine with 0.01 mg/mL BP9, 0.05 mg/mL BP9, or 0.25 mg/mL BP9. Each mouse was intraperitoneally injected with 200 µL of the assigned treatment. A booster dose was given at 14th days following the initial immunization to detect the antibody level in the immunized groups.

### 2.6. Serum-Specific Antibody Level Detection

The indirect ELISA method was used to determine the antibody levels in the sera collected at 7th day after the second immunization. Ninety-six-well ELISA plates were coated with 2 µg/mL of purified inactivated virus as antigen [7]. Subsequently, after blocking, sera sample incubation and secondary antibody addition (PK20003, Proteintech, Wuhan, China), TMB substrate colonization were performed according to standard ELISA procedures. Finally, the microplate was measured at the optical density (OD) values at a 450 nm wavelength to analyze the antibody level in the immunized mice.

### 2.7. MTT Assay/Splenic Lymphocyte Activity Assessment

Lymphocytes were isolated using an appropriate quantity of magnetic beads. Approximately 10^5^ cells per well were cultivated into 96-well cell culture plates, in which PBS (pH = 7.4) control was the media control. All splenic lymphocytes from five immunized groups were treated with 10 ng/mL LPS. After 48 h, 20 µL of MTT was added to each well for 4 h. The culture medium was discarded, and 100 µL of DMSO was added to each well. The OD values of cell plates were measured at 570 nm to determine the lymphocyte viability [9,17].

### 2.8. FCM

The spleen lymphocytes collected from the immunized mice were incubated with FITC, PE, Alexa Fluor, and PerCP-Cy5 labeled CD19 (553758, BD Biosciences), CD27 (558754, BD Biosciences), CD38 (562769, BD Biosciences), and CD69 (551113, BD Biosciences) antibodies for 30 min. After washing, the labeled lymphocytes were analyzed with flow cytometry to identify the different subtypes of B cells in the different immunization groups [7,9].

### 2.9. RNA Sequencing of B-Cells

The spleen-derived suspension of lymphocytes was mixed with the recommended amount of magnetic bead antibodies for B-cell sorting [9]. After washing and centrifugation at 300 g for 7 min, the spleen lymphocytes were incubated with the CD45R/B220 magnetic bead (551513, BD Biosciences) addition at 4 °C for 30 min, and the mixture was transferred to a flow tube. This sorting process was repeated twice to screen the B cells.

The screened cells were used for RNA sequencing, which was accomplished in the Tsingke Biotechnology Company (Beijing, China). The procedure was as reported in a previous method [8]. Simply, the total RNA was extracted from each group’s samples using Trizol method, and RNA quantity was assessed using the ND-2000 (Nanodrop) and agarose gel electrophoresis method, ensuring that the sample purity and integrity were suitable for analysis (RIN ≥ 8). Then, the total RNA samples were subjected to mRNA enrichment using Oligo (DT) magnetic beads. The enriched mRNA was fragmented and subjected to reverse transcription using random primers for end repair. Finally, sequencing was conducted. To analyze the differential expressions of genes between the BP9 combined vaccine immunization group and the vaccine control group, the expression levels of each transcript were calculated based on the method of fragments per kilobase of transcript per million mapped reads (FPKM). The parameter thresholds for significantly different genes were |log2 fold change| ≥ 1 and q < 0.05.

Additionally, the functional enrichment analysis was carried out in database of Gene Ontology (GO) and the Kyoto Encyclopedia of Genes and Genomes (KEGG). Significantly differentially expressed genes (DEGs) enriched in the GO terms and metabolic pathways were identified when the q-value (*p*-value after correction) was ≤0.05 in comparison with the control group for both GO functional enrichment and KEGG pathway analysis.

### 2.10. HD11 Cell Treatment and Detection

HD11 cells were treated with BP9 from 0.01 to 100 μg/mL for 24 h, and the viabilities were measured with the MTT assay [9,17]. Also, at 6, 12, 24, and 48 h after incubation, the total RNA samples of HD11 cells treated with BP9 were collected to detect the cytokine levels of IL-1β, IL-6, IL-10, iNOS, and IFN-α with qPCR. The sequences of five cytokines were obtained from the NCBI reference sequence, and the designed primers of all cytokines are listed in Table 1, where β-actin was used as the reference gene.

### 2.11. Sample Preparation and miRNA Sequencing

The total RNAs of HD11 cells treated with 100 ng/mL BP9 and 10 ng/mL BSA for 24 h were collected with Trizol to high-throughput miRNA sequencing, which was conducted by the Novogene Company (Beijing, China). The procedure was as reported in a previous method [9,18,19]. Simply, after quality qualification of total RNA and cDNA library construction, the biological information was analyzed based on the reference genome of the chicken species. The volcano plot was used to infer the overall distribution of differential miRNAs, which were evaluated at two levels, namely fold change and corrected significance level (padj/qvalue). Based on the correspondence between miRNA and its target genes (http://www.mirbase.org/, 23 August 2019), GO and KEGG pathways were performed on the set of target genes with differential expressions of miRNA in each group.

### 2.12. Verification of DEGs in HD11 Cells with BP9 Treatment

HD11 cells were treated with 100 ng/mL BP9 and 10 ng/mL BSA for 24 h, and total RNA samples were collected [9]. Three differential miRNAs were randomly selected to be verified with the miRNA qPCR Assay Kit (CWBIO, Nanjing, China), where the forward primers for selected differential miRNAs were designed, as shown in Table 2, and 5sR-F was used as control. The downstream primers were provided in the qPCR assay kit, and the qPCR was performed according to the procedure. The expression level was calculated using the 2^−ΔΔCt^ method.

### 2.13. Data Statistics and Analysis

Data were compiled and visualized with One-way ANOVA using Prism GraphPad 6.0 software, which presented the experimental results as mean ± standard deviation error. * *p* < 0.05; ** *p* < 0.01; *** *p* < 0.001.

## 3. Results

### 3.1. BP9 Enhanced the Antibody Production and the Proliferation of Splenic Lymphocytes In Vivo

**Figure 1:**
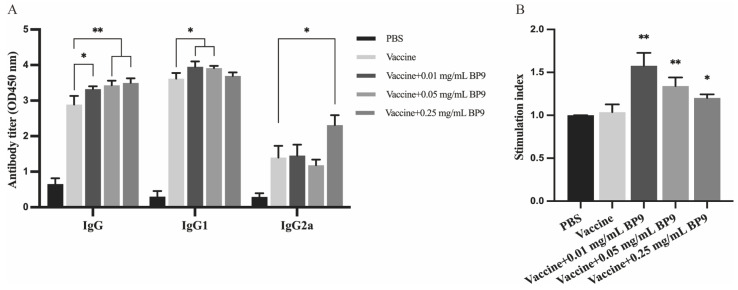
BP9 induced antibody production and lymphocyte viability proliferation. BALB/c mice were immunized with vaccine and BP9 at three dosages. After the second immunization, the antibody levels and lymphocyte viabilities were detected. (**A**) The effect of BP9 on the level of antibodies. (**B**) The effect of BP9 on the proliferation of splenic lymphocytes. * *p* < 0.05, ** *p* < 0.01 compared to that of vaccine control.

In order to study the effects of the bursal-derived peptide on antibody production and B cells, mice were immunized with AIV inactivated vaccine combined with three different concentrations of bursal peptide BP9 (0.01, 0.05, and 0.25 mg/mL) as an immunization model. As shown in Figure 1A, compared with the vaccine control group, all three doses of BP9 significantly promoted the increase in IgG levels in mice, where 0.01 and 0.05 mg/mL BP9 significantly stimulated an increase in IgG1 levels in mice, while 0.25 mg/mL BP9 significantly increased IgG2a levels in mice. These results suggested that BP9 should be involved in regulating humoral immune responses in vivo.

Furthermore, the MTT colorimetric method was used to detect the proliferation of splenic lymphocytes in the immunized mice. As shown in Figure 1B, compared with the vaccine control group, the viabilities of splenic lymphocytes in the group with three doses of BP9 immunization were significantly increased, where 0.01 mg/mL BP9 immunization group showed the most significant cell proliferation activity, indicating that BP9 could stimulate splenic lymphocyte proliferation in vivo.

### 3.2. BP9 Stimulated the Differentiation of B Cells In Vivo

**Figure 2:**
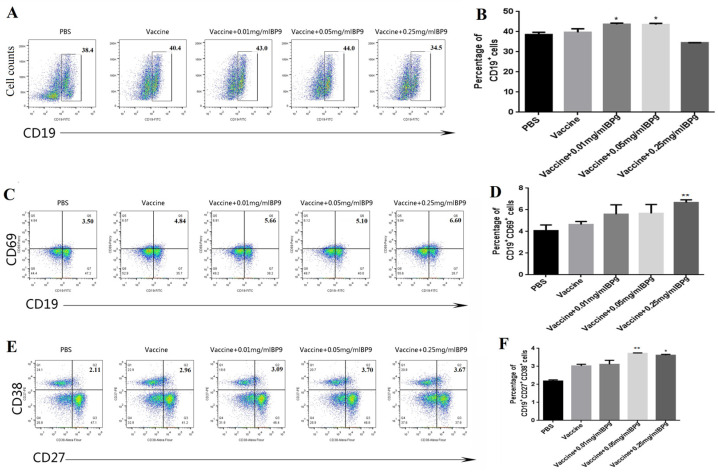
BP9 induced the differentiation of B cells in immunized mice in vivo. BALB/c mice were immunized with vaccine and BP9, and differentiations of B cells were detected at day 7 after the second immunization. (**A**) Example spots of CD19+ total cells in each group. (**B**) Percentage of CD19+B cells. (**C**) Examples of CD19–CD69 labeled flow scatter graphs in each group. (**D**) Percentage of CD19+CD69+B cells. (**E**) Examples of CD27–CD38 labeled flow scatter graphs in each group. (**F**) Percentage of CD19+CD27+CD38+B cells. The horizontal arrow indicates that the horizontal axis of each group is the same. * *p* < 0.05, ** *p* < 0.01, compared to that of vaccine control.

In order to investigate the effect of BP9 on total B cells, the spleen lymphocytes from the immunized mice were incubated with CD19-FITC labeled antibodies and were analyzed with FCM. The flow cytometry plots of CD19+B-cell percentages are shown in Figure 2A. Compared with the vaccine group, the percentages of total CD19+B cells in mice immunized with 0.01 mg/mL and 0.05 mg/mL BP9 were significantly increased (Figure 2B, *p* < 0.05), suggesting that BP9 might promote total B-cell production. However, the population of total B cells in the 0.25 mg/mL BP9 group was decreased, indicating that BP9 might have duality, namely promotion at low concentrations and inhibition at high concentrations.

To investigate the effect of BP9 on B-cell activation, the spleen lymphocytes were incubated with FITC and Percy labeled CD19 and CD69 antibodies for detection with FCM. As shown in Figure 2C,D, the percentages of CD19+CD69+ activated B cells in the vaccine immunization groups combined with BP9 in a dose-dependent manner were higher than those in the vaccine control group. Also, the percentage of B cells in the 0.25 mg/mL BP9 combined with vaccine immunization group was the highest among all the immunization groups. These results suggested that BP9 should promote B-cell activation.

Plasma cells are the functional B cells for producing antibodies. In order to verify the effect of BP9 on plasma cells in vivo, the spleen lymphocytes were incubated with FITC, PE, and ALexa Flower labeled CD19, CD27, and CD38 antibodies for verification with FCM. The results showed that the plasma cell levels (CD19+CD27+CD38+) in the 0.05 mg/mL and 0.25 mg/mL combination vaccine immunized groups were higher than those in the vaccine control group, where the ratio of plasma cells in the 0.05 mg/mL BP9 immunization group was the highest (Figure 2E,F). The results showed that BP9 should promote the differentiation of plasma cells.

### 3.3. BP9 Induced Differential Expressions of Multiple Genes and Transcription Factors

In order to gain a deeper understanding of the molecular mechanism by which bursal-derived active peptides regulate the development of B cells, the samples of B cells from mice immunized with 0.01 mg/mL BP9 and the vaccine were purified using B220 magnetic beads. The purity of B cells was up to 96.7%, which met the requisite experimental standards.

#### 3.3.1. Statistical Assessment of Differentially Expressed Genes

**Figure 3:**
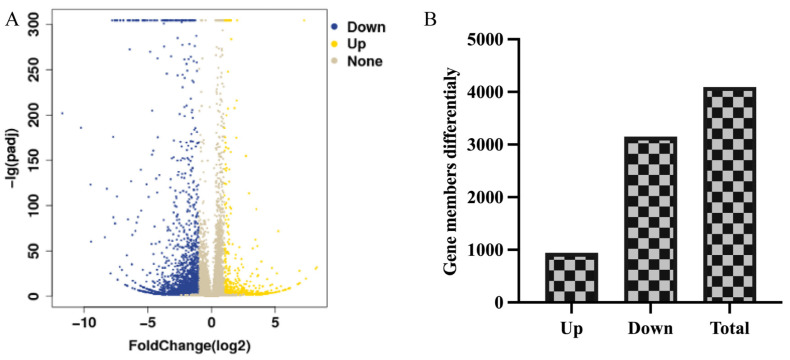
DEGs of B cells induced with BP9 immunization. B cells were isolated from the mice immunized with 0.01 mg/mL BP9 and the vaccine to detect the gene expression profiles using RNA sequencing. (**A**) The volcano plot of regulated genes in B cells. Blue: the down-regulated genes with significant differences; Yellow: the up-regulated genes with significant differences; Brown: the genes without significant differences. (**B**) The number of DEGs in B cells.

The gene expression profiles in 0.01mg/mL BP9 immunization group were comparatively analyzed with the vaccine control to determine the number of differentially expressed genes (DEGs). The volcano plot of the DEGs was displayed in Figure 3A, and there were 4093 DEGs in 0.01 mg/mL BP9 immunized group, where 943 DEGs were upregulated, and 3150 DEGs were downregulated (Figure 3B). Detailed information about the DEGs was listed in Supplementary Material S1, which was used for the functional enrichment analysis.

#### 3.3.2. BP9 Enhanced Various Antigen Presentation Pathways

In order to analyze the molecular mechanism of BP9 on B cells activation, the KEGG Pathway tool was used to investigate the enriched signal pathway of DEGs in the BP9 immunization group (Table 3), whose detailed information is listed in Supplementary Material S2. The significantly enriched pathways included the interaction between cytokines and cytokine receptors, ECM–receptor interaction, cell adhesion molecules (CAMs), and complement and coagulation cascades. In addition, various metabolic pathways, such as protein digestion and absorption, tryptophan metabolism, serine and threonine metabolism, glyceride metabolism, porphyrin and chlorophyll metabolism, and glutathione metabolism, were also significantly activated. These results suggested that BP9 could activate various immune-related pathways within B cells.

#### 3.3.3. BP9 Activated the Multiple Biological Processes in B Cells

The immune-related functional processes play important roles in B-cell activation and humoral immunity. To investigate the functional mechanism of BP9 in B cells, the GO was used to analyze the biological significance of DEGs in B cells from mice immunized with BP9 and the vaccine, where the immune-related biological processes (Supplementary Material S3) were emphasized for analysis. It was observed that there were thirteen immune-related processes, five immune-related signal transductions, nine B-cell-related processes, eleven lymphocyte- and T-cell-related processes, and twenty-five cytokine-related processes. Furthermore, based on molecular function analysis (Supplementary Material S4), the enriched terms included immunoglobulin receptor binding, antigen binding, and eight cytokine- and chemokine-related molecular functions. Additionally, the enriched cellular components were two immunoglobulin complex terms (Supplementary Material S5). These results suggested that BP9 could activate multiple immune-related functional processes in B cells, which might lead to the differentiation of B cells and humoral immune responses in immunized mice.

#### 3.3.4. BP9 Activated the Multiple Transcript Factors in B Cells

**Figure 4:**
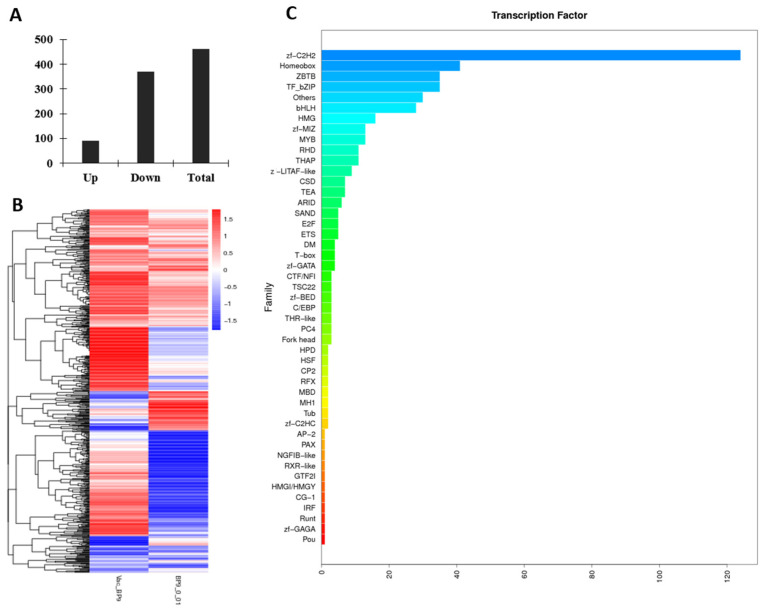
BP9 induced various differentially expressed transcription factors in B cells in vivo. (**A**) The differentially expressed transcription factors. (**B**) The heatmap analysis of transcription factors. (**C**) The families of the transcription factors in B cells in vivo.

The differentiation of B cells is closely related to transcription factors. In this study, the expression profiles of transcript factors in B cells from mice immunized with BP9 and the vaccine were detected (Supplementary Material S6). As shown in Figure 4, there were 90 upregulated genes and 371 downregulated genes in the differentially expressed transcription factors in B cells after BP9 immunization (Figure 4A), whose heatmap analyses are listed in Figure 4B. Furthermore, the families involved in the differentially expressed transcription factors were displayed in Figure 4C. It was observed that the number of transcription factors involved in the zf-C2H2 family was the highest, which was more than 120 transcription factors. Additionally, families with more than 20 transcription factors included the Homeobox, ZBTB, TF_bZIP, and bHLH families. These results suggested that BP9 might regulate various transcription factors, which lead to B cell differentiation and maturation.

### 3.4. BP9 Stimulated the Cytokine Expressions in Chicken Macrophages

**Figure 5:**
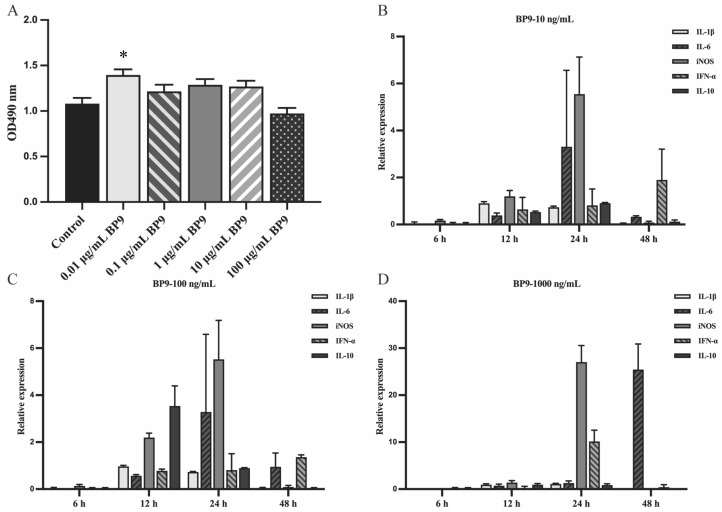
Effect of BP9 on viabilities and cytokines in macrophage cells. HD11 cells were treated with BP9 at different concentrations, and the viabilities and cytokines at different times were detected. (**A**) Viabilities with BP9 treatment. * *p* < 0.05, compared to control. (**B**–**D**) Cytokine mRNA expressions of macrophages stimulated with different concentrations of BP9 at different times. (**B**) 10 ng/mL BP9. (**C**) 100 ng/mL BP9. (**D**) 1000 ng/mL BP9.

To investigate the molecular mechanism of BP9 in antigen presentation in chicken-derived cells, the chicken macrophage HD11 cells were used to detect viability after BP9 treatment. It was observed that compared with the control group without BP9 treatment, BP9 at experimental concentrations enhanced the proliferation of HD11 cells, where the proliferation ability of HD11 with 0.01 μg/mL BP9 treatment was significantly increased and that of 100 μg/mL BP9 treatment was similar to control (Figure 5A). These results suggested that BP9 within a certain concentration range might promote the viability of chicken macrophage HD11 cells.

In order to explore the regulatory mechanism of BP9 in macrophage function, a quantitative PCR was used to analyze the mRNA expression levels of five representative factors with BP9 treatment. The results showed that the expression levels of IL-1β, IL-6, iNOS, IFN-α, and IL-10 were increased after 10 ng/mL BP9 stimulation at 24 h (Figure 5B), where the levels of IL-6 and iNOS were higher than those of the three factors. Surprisingly, the levels of IL-1β, IL-6, iNOS, IFN-α, and IL-10 were significantly increased after 100 ng/mL BP9 treatment at 12 and 24 h, and the expression levels of cytokines at 24 h were higher than those at 12 h (Figure 5C). However, the expression levels of iNOS and IFN-α in 1000 ng/mL BP9 treatment group were increased at 24 h, and only iNOS was upregulated at 48 h (Figure 5D). It could be inferred from these results that the changes in various functional factors might be significant with the 100 ng/mL BP9 treatment at 24 h, and these cell samples would be selected as the samples for subsequent high-throughput data analysis.

### 3.5. BP9 Promoted Various Signal and Biological Processes in Chicken Macrophages

**Figure 6:**
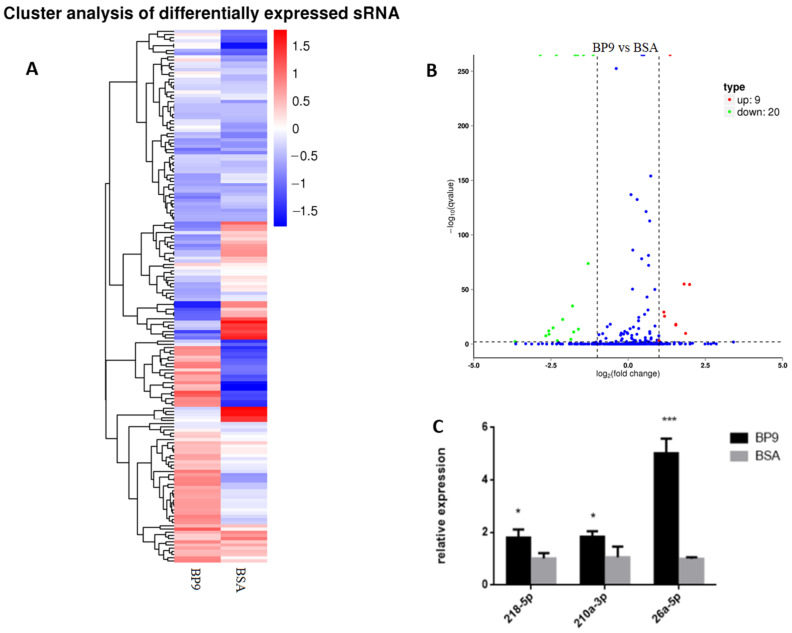
The miRNA expression profiles of HD11 cells with BP9 treatment. (**A**) The overall hierarchical clustering diagram of differentially expressed miRNAs. The clustering diagram was clustered based on log10 (TPM + 1) values. (**B**) The differential miRNA volcano map. The scatter dots in the figure represent various differential miRNAs, where the blue dots represent miRNAs with no significant differences; the red dots represent the significantly upregulated differential miRNAs; and the green dots represent the significantly downregulated differential miRNAs. (**C**) Real-time quantitative PCR verification of BP9 differentially expressed miRNAs. * *p* < 0.05, *** *p* < 0.001, compared to that of BSA.

The RNA samples from HD11 cells treated with 100 ng/mL BP9 for 24 h were analyzed to investigate the miRNA expression profiles, which was showed in Supplementary Material S7, and the overall hierarchical clustering diagram of differentially expressed miRNAs was shown in Figure 6A. There were 9 upregulated differential miRNAs and 20 downregulated differential miRNAs, whose volcano map of differential miRNAs is shown in Figure 6B. Also, some miRNAs from differentially expressed miRNAs were validated with quantitative PCR (qPCR), and three miRNAs (218-5p, 210a-3p, and 26a-5p) were upregulated (Figure 6C), which were similar to those of the miRNA expression profiles. These results suggested that BP9 might regulate various miRNAs expression in chicken macrophages.

To investigate the pathways of BP9 in chicken macrophages, the enriched pathways included cell adhesion molecules (CAMs), the intestinal immune network for IgA production, Notch signaling pathway, phagosome, and ribosome (Supplementary Material S8). Furthermore, the GO process in which BP9 induced immune-related processes in HD11 cells mainly focused on antigen presentation, T cells, and cytokines (Supplementary Material S9). It was observed that BP9 stimulated various MHC-related cellular functions, such as the MHC protein complex and antigen processing and presentation. Also, BP9 enhanced four T-cell-related biological processes and three cytokine-related biological processes. Additionally, there were two DEGs involved in the macrophage migration inhibitory factor signaling pathway in HD11 cells treated with 100 ng/mL BP9. These results suggested that BP9 might activate various antigen-processing- and presentation-related biological functions, which lead to immune enhancement.

## 4. Discussion

The avian immune system constitutes a critical determinant in the efficacy of resistance toward pathogenic micro-organisms [20,21,22]. B-cell development, maturation, and function play vital roles during humoral immunity, which is important to the immune enhancement and disease prevention and control [23]. Further research into the immune functions of BF in B-cell maturation and antibody generation holds potential for enhancing the understanding of immune signaling and induction pathways upon antigen provocation [24,25]. Insights into the extracellular regulatory mechanisms of B cells in BF, however, remain somewhat elusive.

The bursal-derived peptide BP9 is a recently reported immunomodulatory peptide that enhances the immune response to inactivated avian influenza vaccines in chickens and mice [14,15]. In this experiment, it was found that BP9 vaccination not only enhanced the increased levels of IgG1 and IgG2a subtype antibodies but also stimulated B-cell activation and plasma cells. These results indicated that BP9 could stimulate B-cell proliferation and activate B cells to differentiate into plasma cells, leading to antibody production.

The development of B cells is a highly controlled process that requires the regulation of multiple important transcriptional factors and gene signals [26]. mRNA is widely distributed in many eukaryotes, especially mammals [27,28]. However, little is known about the regulation of mRNA in B-cell development by BF. In order to explore the mechanism of the bursal active peptide regulating B-cell development in vivo, in this paper, RNA sequencing technology was used to detect mRNA differences in a group immunized with 0.01 mg/mL BP9 combined with the avian influenza vaccine. It was found that there were 4093 DEGs in the BP9 group. The functional processes of DEGs were further analyzed with KEGG and GO databases. The enriched pathways showed that BP9 stimulated various cytokines, complement, adhesion molecules, and metabolic-related pathways. Also, the GO classification analysis revealed that BP9 induced various immune signal transductions, B-cell-, T-cell-, cytokine-related biological processes, and it stimulated immunoglobulin receptor and binding, antigen binding, and cytokine- and chemokine-related molecular functions.

Additionally, BP9 induced 461 differentially expressed transcription factors in B cells, which were involved in various transcription factor families, especially the C_2_H_2_ family. Transcription factors play an important role in the development of B cells. During the pathogenesis of GC-derived lymphomas, the mutation of the transcription factor forkhead box O1 (FOXO1) enables co-option of GC-positive selection programs [29]. Also, to produce antibodies and differentiate into plasma and memory cells, the activated B cells in GC undergo a range of clonal expansions and functional maturations, where Ascl2 regulates the AID transcription and promotes GC B-cell responses [30,31,32,33]. These results indicated that bursal active peptides could regulate various mRNA gene levels in regulating B-cell development, which provides the ideas for studying BF regulating B-cell development at the gene level and provides the important experimental basis for bursal active peptides as novel immune enhancers to improve vaccine immunity.

In previous studies, BP9 was found to promote the immune response in immune chickens [15]. In this paper, it was observed that BP9 induces the differentiation and maturation of mouse B cells and activates various biological functions of B cells in immunized mice. However, the mechanism by which BP9 regulates the antigen presentation and immune function of avian-derived cells is still unclear.

Macrophages possess the function of antigen-presenting cells and are important objects in studying cellular immunity and molecular immunology [9,34]. In order to investigate the mechanism of bursal active peptides regulating antigen presentation, in this study, chicken macrophage HD11 cells were used as a model. It was found that 100 ng/mL BP9 strongly stimulated various cytokine expressions. miRNA plays an important role in biological processes, such as tissue development, B-cell receptor signaling [35], proliferation, and apoptosis, as well as virus gene expression [36]. However, the mechanism by which miRNA participates in the regulation of immune response by bursal active peptides is still unclear. In order to identify the key miRNAs that regulate the immune response through regulation of bursal active peptides, the miRNA expression profile of chicken macrophages with BP9 treatment was explored. There were 9 upregulated miRNAs and 20 downregulated miRNAs in HD11 cells with BP9 treatment. These functional analyses showed that BP9 induced various enriched pathways, including CAMs, the intestinal immune network for IgA production, Notch signaling pathway, phagosome, and ribosome. Furthermore, BP9 activated multiple immune-related functional processes, including cytokine, chemokine, T cell, and antigen presentation. The regulation of B-cell development in vivo by bursal active peptides is a complex process that requires the regulation of multiple signals. miRNAs are recognized as pivotal regulators in B-cell responses, integral to both non-autoimmune and vaccine-induced humoral immunity, where they modulate B-cell functions without triggering autoimmune diseases and are instrumental in targeted immune defense strategies [37]. The research results lay the foundation for the subsequent screening of key small RNAs and further research on the functional mechanism of peptide regulation of chicken macrophages, which provides the experimental basis for the clinical vaccine application research of bursal active peptides.

Additionally, a comparative analysis of the enrichment pathways revealed that only the cell adhesion molecules (CAMs) pathway was shared in mouse B cells and chicken macrophages following BP9 treatment. However, gene ontology analysis found that the differentially expressed genes involve multiple similar biological functions in both conditions. B cells not only play an important role in the production of antibodies during vaccine immunization, but they also act as antigen-presenting cells and participate in the process of presenting immune antigens during the immune response [38,39]. The immune system of poultry has unique characteristics and has properties similar to those of mammals [40]. These results suggested that due to differences in cell species and types, the differentially expressed genes induced by BP9 stimulation might participate in different signaling pathways, but the regulation of B-cell function would be similar. These data also suggested that BP9 might induce humoral immunity in different species of animals, which could provide a research basis for the development of a broad-spectrum peptide adjuvant.

## 5. Conclusions

B-cell differentiation and maturation are regulated by multiple gene signals and transcriptional factors. BP9 is a recently reported bursal-derived immunomodulatory peptide with the function of immune response enhancement during vaccine immunization. In this study, BP9 stimulated B-cell activation and differentiation in vivo, and it induced various enriched pathways in the B cells of immunized mice. Also, BP9 activated multiple immune-related functional processes and transcriptional factor families in B cells in vivo. Furthermore, BP9 stimulated various cytokine expressions in chicken macrophages and induced 9 upregulated and 20 downregulated differential miRNAs, which were involved in five signal and various functional processes, including antigen presentation, as well as the MHC protein complex and binding. These results provide valuable insights into the B-cell functional mechanism of bursal-derived bioactive peptides, and they provide a solid experimental groundwork for improving vaccine strategies and developing new adjuvants.

## Figures and Tables

**Table 1:** The designed primers used for qPCR.

Primer Name	NCBI Reference	Sequence (5′-3′)
IL-1β-F	NM_204524.2	ACCCGCTTCATCTTCTACCG
IL-1β-R		TCAGCGCCCACTTAGCTTG
IL-6-F	NM_204628.2	AGGACGAGATGTGCAAGAAGTTC
IL-6-R		TTGGGCAGGTTGAGGTTGTT
IL-10-F	NM_001004414.4	CGCTGTCACCGCTTCTTCA
IL-10-R		CGTCTCCTTGATCTGCTTGATG
iNOS-F	U46504.1	AGGCCAAACATCCTGGAGGTC
iNOS-R		TCATAGAGACGCTGCTGCCAG
IFN-α-F	GU119896.1	GGACATGGCTCCCACACTAC
IFN-α-R		GGCTGCTGAGGATTTTGAAGA
β-actin-F	NM_205518.2	AGACATCAGGGTGTGATGGTTGGT
β-actin-R		TGGTGACAATACCGTGTTCAATGG

**Table 2:** Primer sequences for verifying DEGs by qPCR.

Primer Name	Sequence (5′-3′)
gga-miR-26a-5p	TCCAGCTGGGTTCAAGTAATCCAGG
gga-miR-210a-3p	CTGTGCGTGTGACAGCGGCTAA
gga-miR-218-5p	TTGTGCTTGATCTAACCATGT
5sR-F	GTCTACGGCCATACCACCCTGAAC

**Table 3:** The enriched pathways in B cells from mice immunized with BP9 and the vaccine.

Name	Q-Value	Up Count	Down Count
Hematopoietic cell lineage	5.0588 × 10^−9^	3	30
Cytokine–cytokine receptor interaction	1.4634 × 10^−8^	13	53
ECM–receptor interaction	6.8286 × 10^−6^	6	20
Cell adhesion molecules (CAMs)	3.2955 × 10^−5^	5	32
Protein digestion and absorption	0.00014185	3	23
Tryptophan metabolism	0.00014185	3	14
ABC transporters	0.00020629	1	17
Malaria	0.00027825	4	13
Steroid biosynthesis	0.00059044	0	10
Glycine, serine, and threonine metabolism	0.00158069	3	17
African trypanosomiasis	0.00225022	3	9
Nitrogen metabolism	0.00231943	2	8
Glycerolipid metabolism	0.0034593	2	18
Osteoclast differentiation	0.00443348	8	21
Axon guidance	0.0054491	8	31
Pathways in cancer	0.005574	14	92
Porphyrin and chlorophyll metabolism	0.00712266	0	13
Glutathione metabolism	0.00831065	2	22
Complement and coagulation cascades	0.00896268	7	14
Ether lipid metabolism	0.00942156	3	12
Valine, leucine, and isoleucine degradation	0.01069113	3	12
Alanine, aspartate, and glutamate metabolism	0.01326763	1	12
Glycerophospholipid metabolism	0.01326763	4	19
Metabolism of xenobiotics by cytochrome P450	0.01326763	2	19
Pancreatic secretion	0.01326763	1	24
cAMP signaling pathway	0.02294556	6	33
Fat digestion and absorption	0.02354943	0	12
MAPK signaling pathway	0.02362328	9	44
microRNAs in cancer	0.025189	6	27
Neuroactive ligand–receptor interaction	0.04037019	12	39
Fluid shear stress and atherosclerosis	0.04050935	7	33
Relaxin signaling pathway	0.04050935	4	22
Mineral absorption	0.04379525	0	13
Bile secretion	0.04465393	0	17
Vitamin digestion and absorption	0.04546349	2	6
Glyoxylate and dicarboxylate metabolism	0.04715224	1	11
Adrenergic signaling in cardiomyocytes	0.04788709	8	21

## Data Availability

The data presented in this study are available upon request from the corresponding author.

## Supplementary Materials

The following supporting information can be downloaded at: https://www.mdpi.com/article/10.3390/vaccines12060607/s1, Supplementary Material S1. The DEGs in B cells from mice immunized with BP9 and the vaccine; Supplementary Material S2. The enriched pathways in B cells from mice immunized BP9 and the vaccine; Supplementary Material S3. The immune-related biological processes involved in DEGs in B cells from mice immunized with BP9 and the vaccine; Supplementary Material S4. The immune-related molecular functions involved in DEGs in B cells from mice immunized with BP9 and the vaccine; Supplementary Material S5. The immune-related cellular components involved in DEGs in B cells from mice immunized with BP9 and the vaccine; Supplementary Material S6. The differential transcription factors in B cells from mice immunized with BP9 and the vaccine; Supplementary Material S7. The differential miRNAs in HD11 cells with BP9 treatment; Supplementary Material S8. The KEGG involved in the differential miRNAs in HD11 cells with BP9 treatment; Supplementary Material S9. The GO involved in the differential miRNAs in HD11 cells with BP9 treatment.
